# Supplementary material for: Some fundamental elements for studying social-ecological co-existence in forest common pool resources
Source: PeerJ. 2023 Feb 27;11:e14731. doi: 10.7717/peerj.14731 (PMC9979833; doi:10.7717/peerj.14731)
Supplement: Supplemental Information 3 — The average timber density of the species considered were extracted from the US forest services (http://www.feis-crs.org/beta/) and various other relevant official sources when not found on the site. [file peerj-11-14731-s003.pdf]

Forest type	Dominant species	Species-level Timber density [kg/m3]	Approximate forest-level timber density [kg/m3]	a1	m1	b1
Spruce/Fir	*Abies balsamea*	400	435	1	1	4.10-4
	Picea rubens	470				
Aspen/Birch	*Populus tremuloides*	450	560	1	0.58	2.8.10-3
	Betula papyrifera	660				
Northern Hardwood	*Betula allaghaniensis*	635	715	1	0.55	3.5.10-3
	Fraxinus americana	675				
	Fagus grandifolia	720				
	Acer saccharum	740				
Oak	*Quercus velutina*	740	760	1	0.4	4.9.10-3
	Quercus prinus	765				
	Quercus alba	770				
