# Supplementary material for: Some fundamental elements for studying social-ecological co-existence in forest common pool resources
Source: PeerJ. 2023 Feb 27;11:e14731. doi: 10.7717/peerj.14731 (PMC9979833; doi:10.7717/peerj.14731)
Supplement: Supplemental Information 5 — The average timber density and the mass of seeds produced of the species considered were extracted from the US forest services (http://www.feis-crs.org/beta/) and various other relevant official sources when not found on the site. [file peerj-11-14731-s005.pdf]

Species name	μ	α	β	γ	Timber density [kg/m3]	Mass of seeds produced [kg]
Abies balsamea	42.54	1.75	0.0014	0.93	400	6.6.10-6
Pinus strobus	43.36	1.02	0.0141	1	410	1.9.10-5
Tilia americana	44.07	1.19	0.0082	1	425	3.1.10-5
Tsuga canadensis	42.2	1.65	0.0011	0.97	450	1.0.10-5
Populus tremuloides	36.73	1.36	0.0064	1	450	1.5.10-7
Picea rubens	39.45	1.85	0.0008	0.89	470	2.3.10-6
Pinus taeda	33.98	1.99	0.0006	0.84	470	2.5.10-5
Liriodendron tulipifera	47.32	2.22	0.0002	0.91	515	3.9.10-5
Liquidambar styraciflua	49.76	1	0.0322	1	545	5.5.10-6
Acer rubrum	42.12	1.92	0.0005	0.94	550	2.0.10-5
Prunus serotina	54.13	2.78	0.001	0.78	595	9.4.10-5
Betula alleghaniensis	45.29	1.45	0.0035	0.99	636	10-5
Betula papyrifera	38.81	1	0.0183	1	660	3.10-7
Fraxinus americana	48.77	2.09	0.0002	0.89	675	3.3.10-5
Quercus rubra	46.25	1.89	0.0005	0.91	670	7.5.10-5
Fagus grandifolia	40.75	1.57	0.0016	0.95	720	2.5.10-4
Acer saccharum	42.12	1.39	0.0035	0.94	740	4.8.10-5
Quercus velutina	46.7	1.48	0.0028	0.91	740	1.9.10-3
Quercus prinus	47.21	2.78	0.00001	0.86	765	4.5.10-3
Quercus alba	47.83	2.8	0.00001	0.88	770	3.8.10-3
Populus grandidentata	34.18	1.45	0.0041	0.99	880	1.8.10-7
