# Supplementary figures and images for: Some fundamental elements for studying social-ecological co-existence in forest common pool resources
Source: PeerJ. 2023 Feb 27;11:e14731. doi: 10.7717/peerj.14731 (PMC9979833; doi:10.7717/peerj.14731)

a)

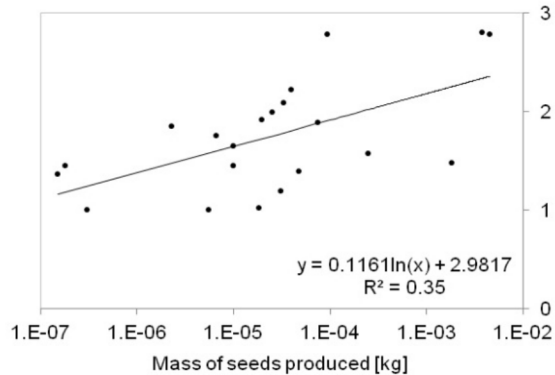

b)

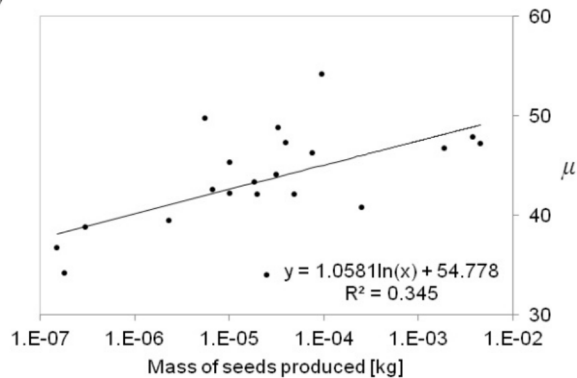

c)

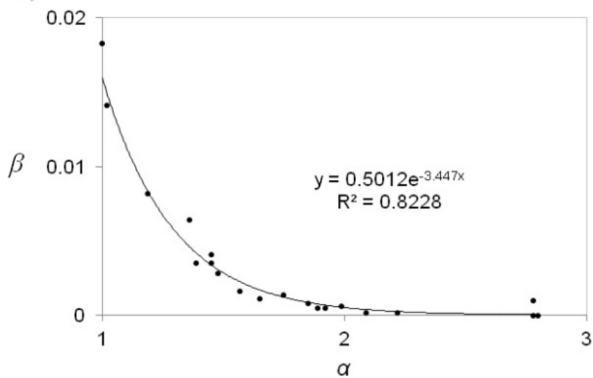

d)

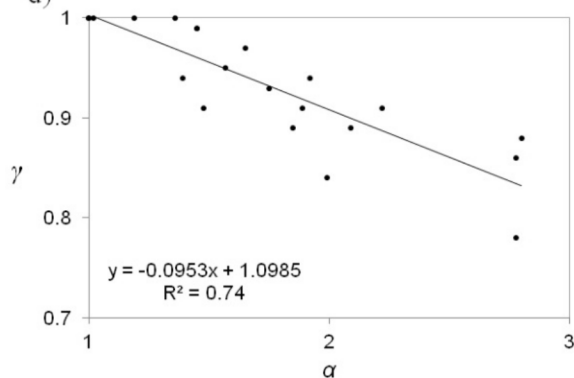

Supplement: Supplemental Information 6 [file peerj-11-14731-s006.pdf]

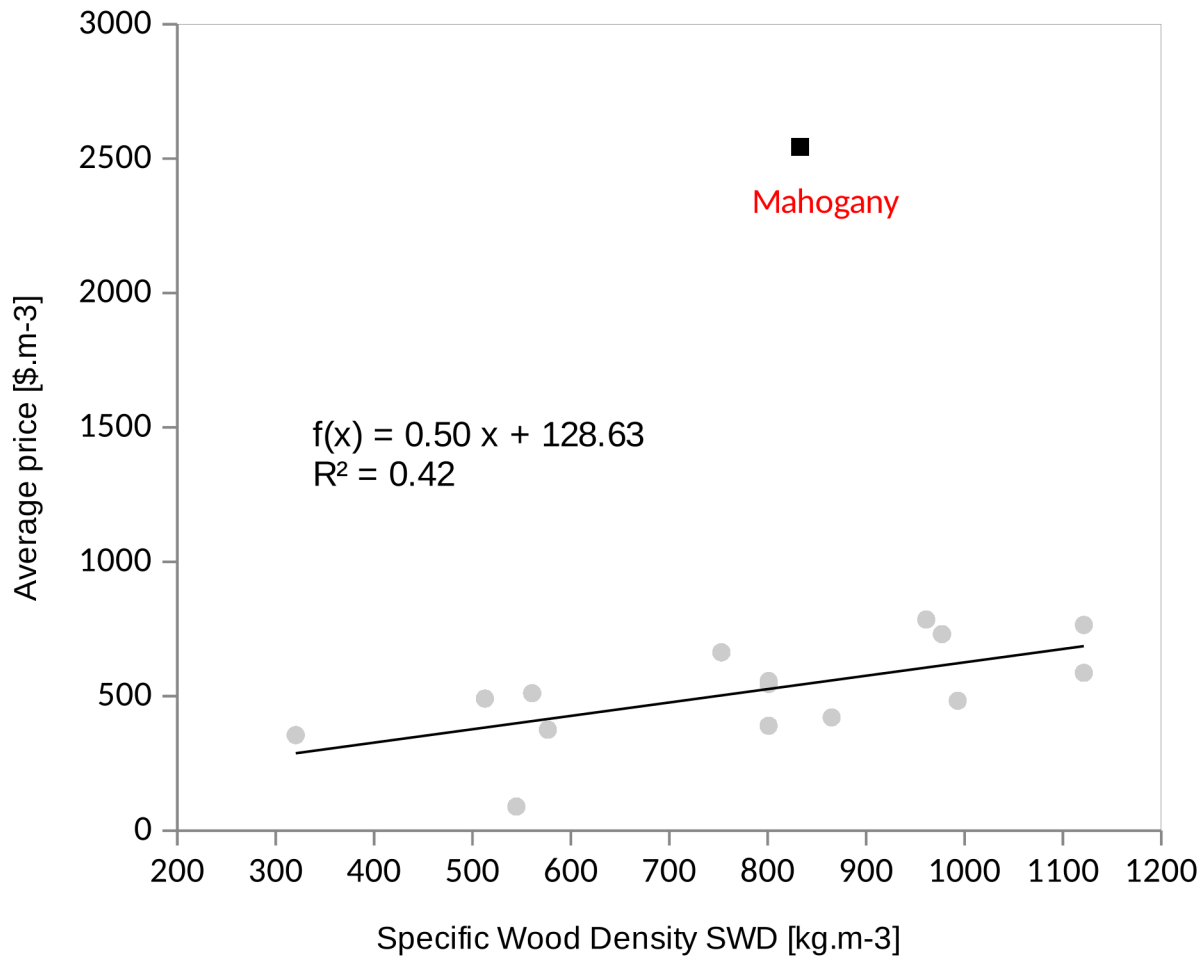

Supplement: Supplemental Information 7 — The outlier represents the Mahogany tree and has been excluded from the regression analysis. Data originally comes from unpublished data used by Ahmed & Ewers (2012), and were graciously provided with permission by Dr. Sadia Ahmed, without indicating the name of the species (except for the outlier mahogany sp.). [file peerj-11-14731-s007.pdf]

0 %

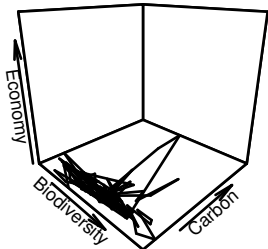

12.5 %

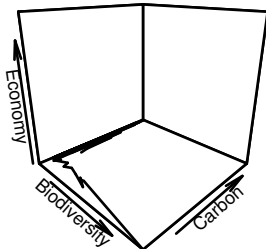

25 %

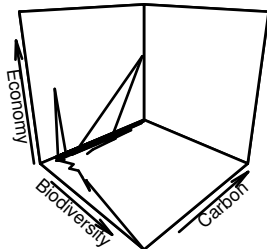

37.5 %

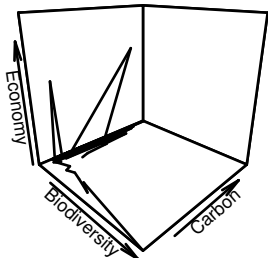

50 %

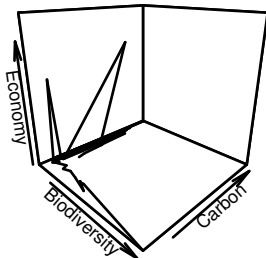

62.5 %

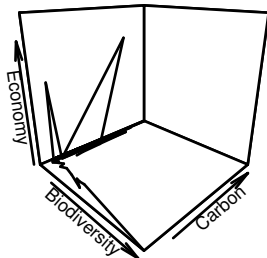

75 %

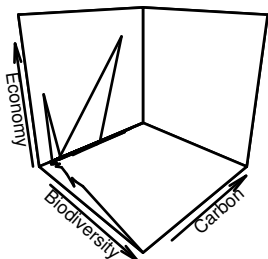

87.5 %

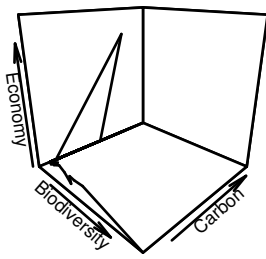

100 %

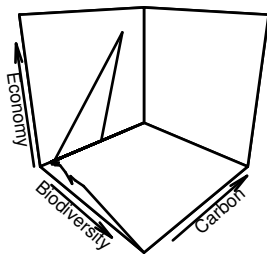

Supplement: Supplemental Information 8 — Annual biodiversity (Shannon index), carbon (AGOC (tonnes CO2 eq . ha−1)) and the economy (collective profit from timber harvesting ($)). For each plot, a line represents the mean trajectories over all the same scenarios with the same proportion of species managed, from 0% to 100%) [file peerj-11-14731-s008.pdf]

0 %

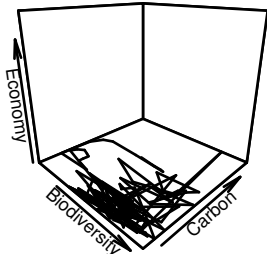

12.5 %

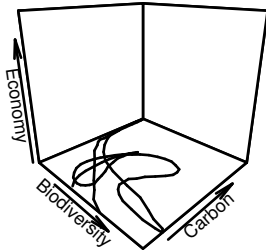

25 %

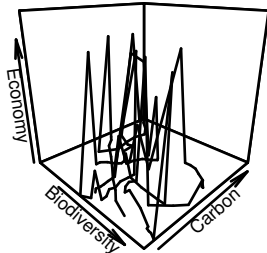

37.5 %

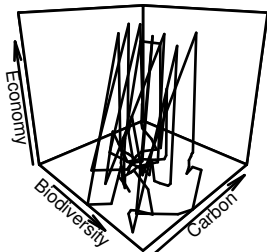

50 %

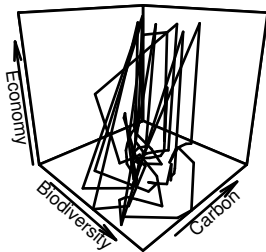

62.5 %

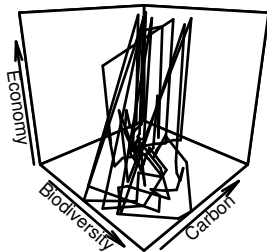

75 %

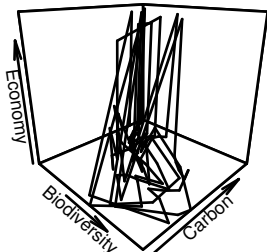

87.5 %

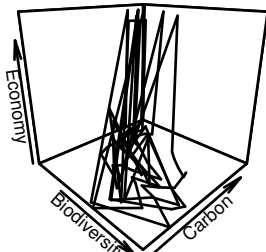

100 %

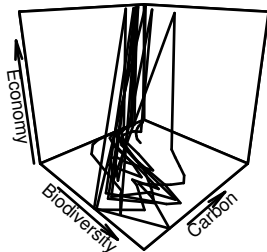

Supplement: Supplemental Information 9 — Annual biodiversity (Shannon index), carbon (AGOC (tonnes CO2 eq . ha−1)) and the economy (collective profit from timber harvesting ($)). For each plot, the line represents the mean trajectories over all the same scenarios with the same proportion of species managed, from 0% to 100%) [file peerj-11-14731-s009.pdf]

0 %

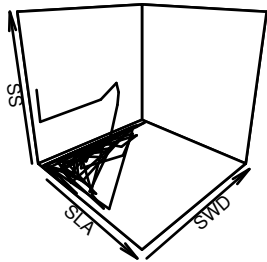

12.5 %

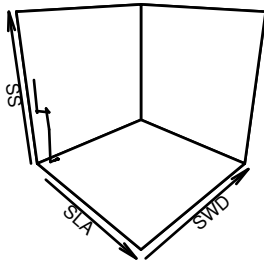

25 %

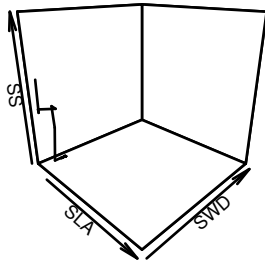

37.5 %

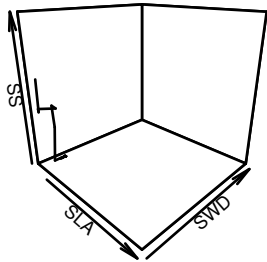

50 %

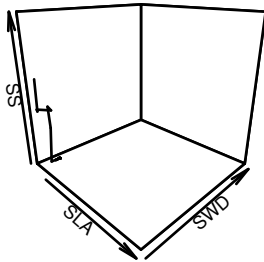

62.5 %

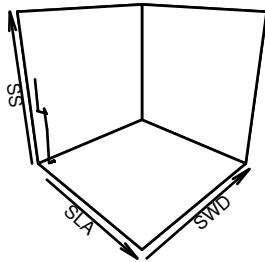

75 %

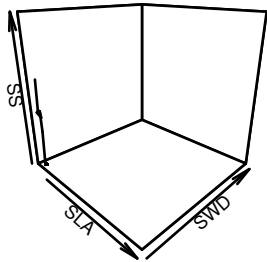

87.5 %

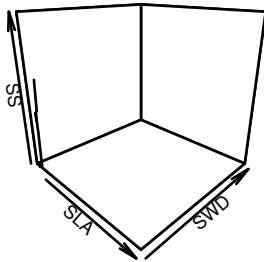

100 %

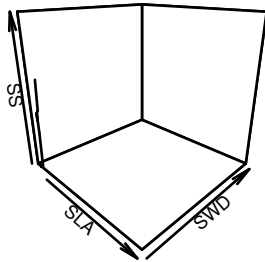

Supplement: Supplemental Information 10 — Specific leaf area SLA (m2.kg−1), specific wood density SWD (kg.m−3), seed size SS (kg) plotted at a log scale. Scatter plots are presented for different proportions of trees managed in drier forest commons containing eight species. For each plot, the line represents the mean trajectories over all the same scenarios with the same proportion of species managed, from 0% to 100%) [file peerj-11-14731-s010.pdf]

0 %

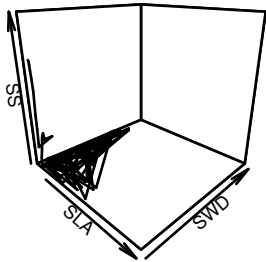

12.5 %

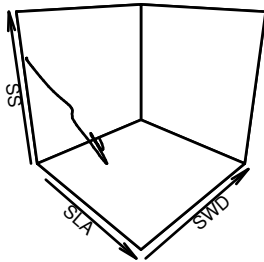

25 %

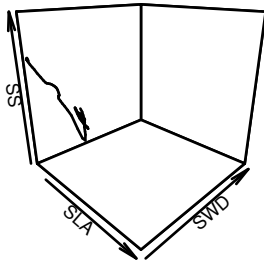

37.5 %

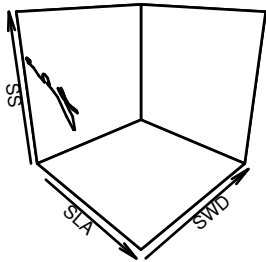

50 %

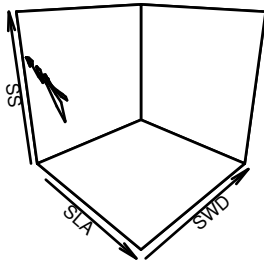

62.5 %

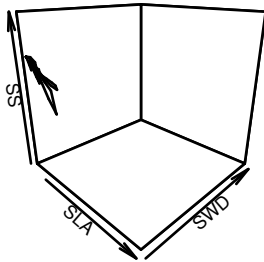

75 %

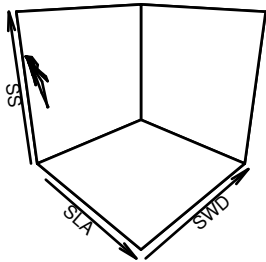

87.5 %

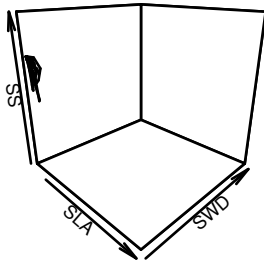

100 %

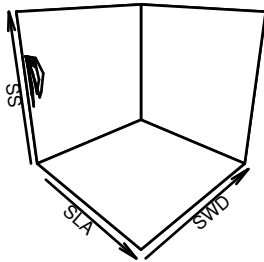

Supplement: Supplemental Information 11 — Specific leaf area SLA (m2.kg−1), specific wood density SWD (kg.m−3), seed size SS (kg) plotted at a log scale. Scatter plots are presented for different proportions of trees managed in wetter forest commons containing eight species. For each plot, the line represents the mean trajectories over all the same scenarios with the same proportion of species managed, from 0% to 100%) [file peerj-11-14731-s011.pdf]

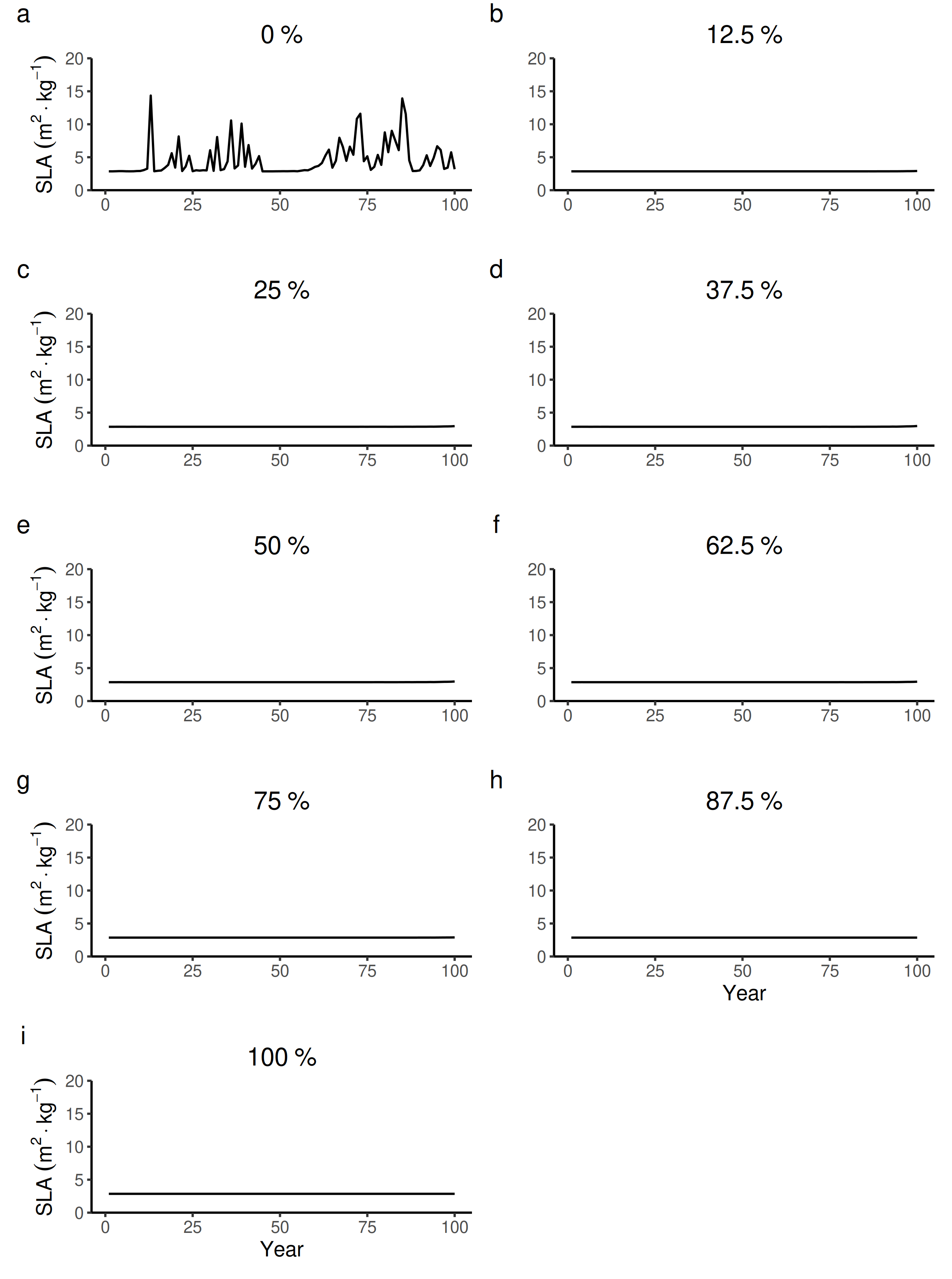

Supplement: Supplemental Information 12 [file peerj-11-14731-s012.png]

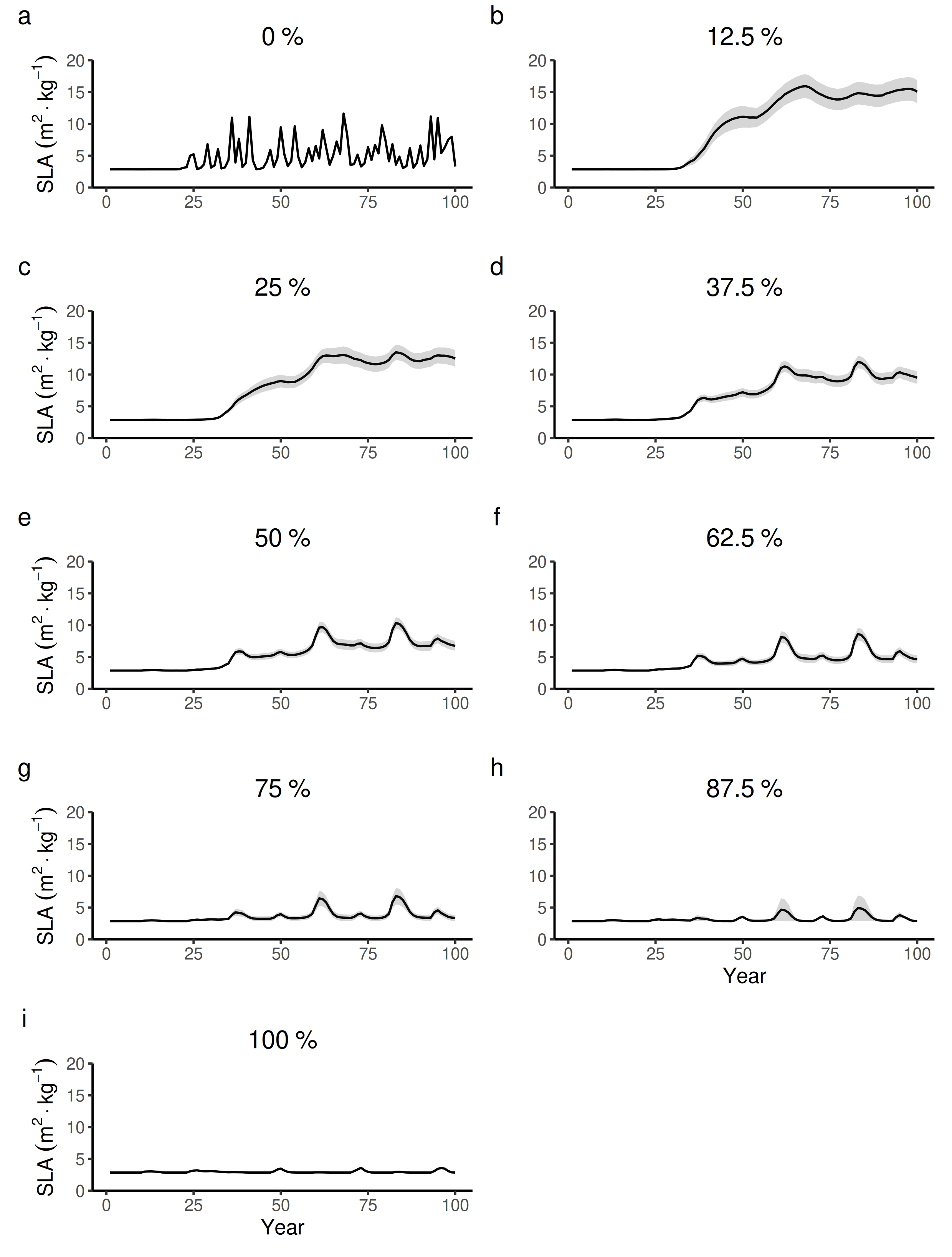

Supplement: Supplemental Information 13 [file peerj-11-14731-s013.png]

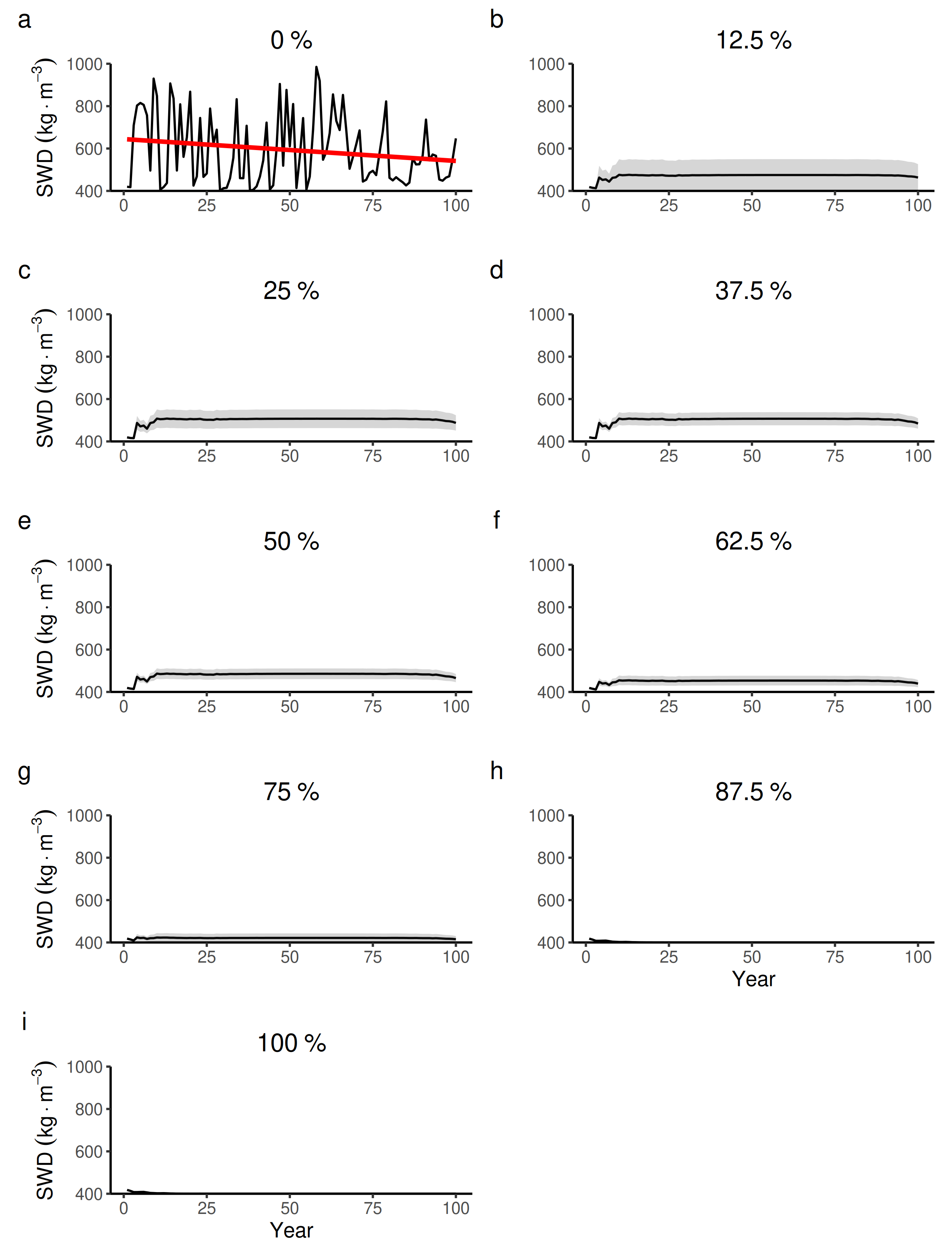

Supplement: Supplemental Information 14 [file peerj-11-14731-s014.png]

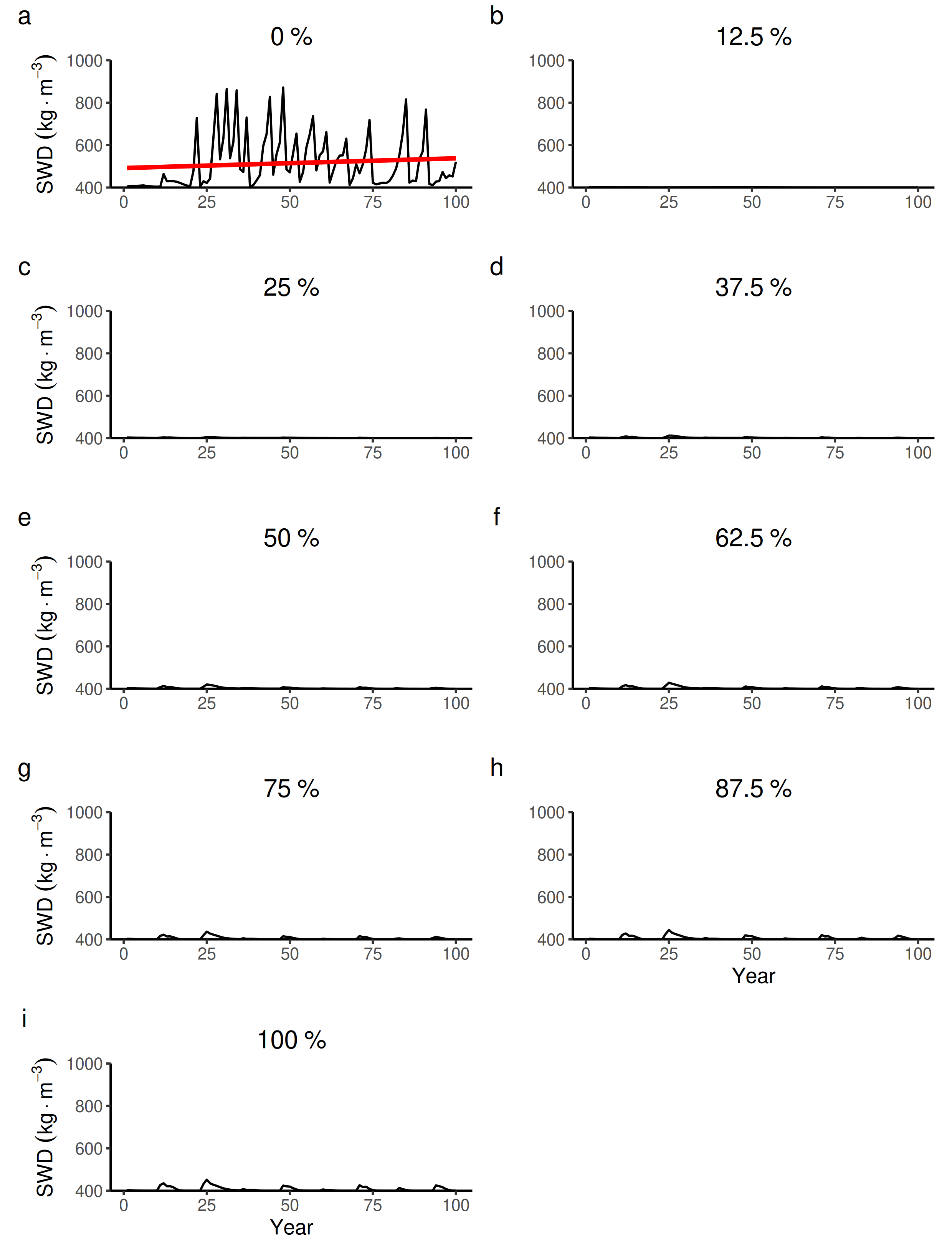

Supplement: Supplemental Information 15 [file peerj-11-14731-s015.png]

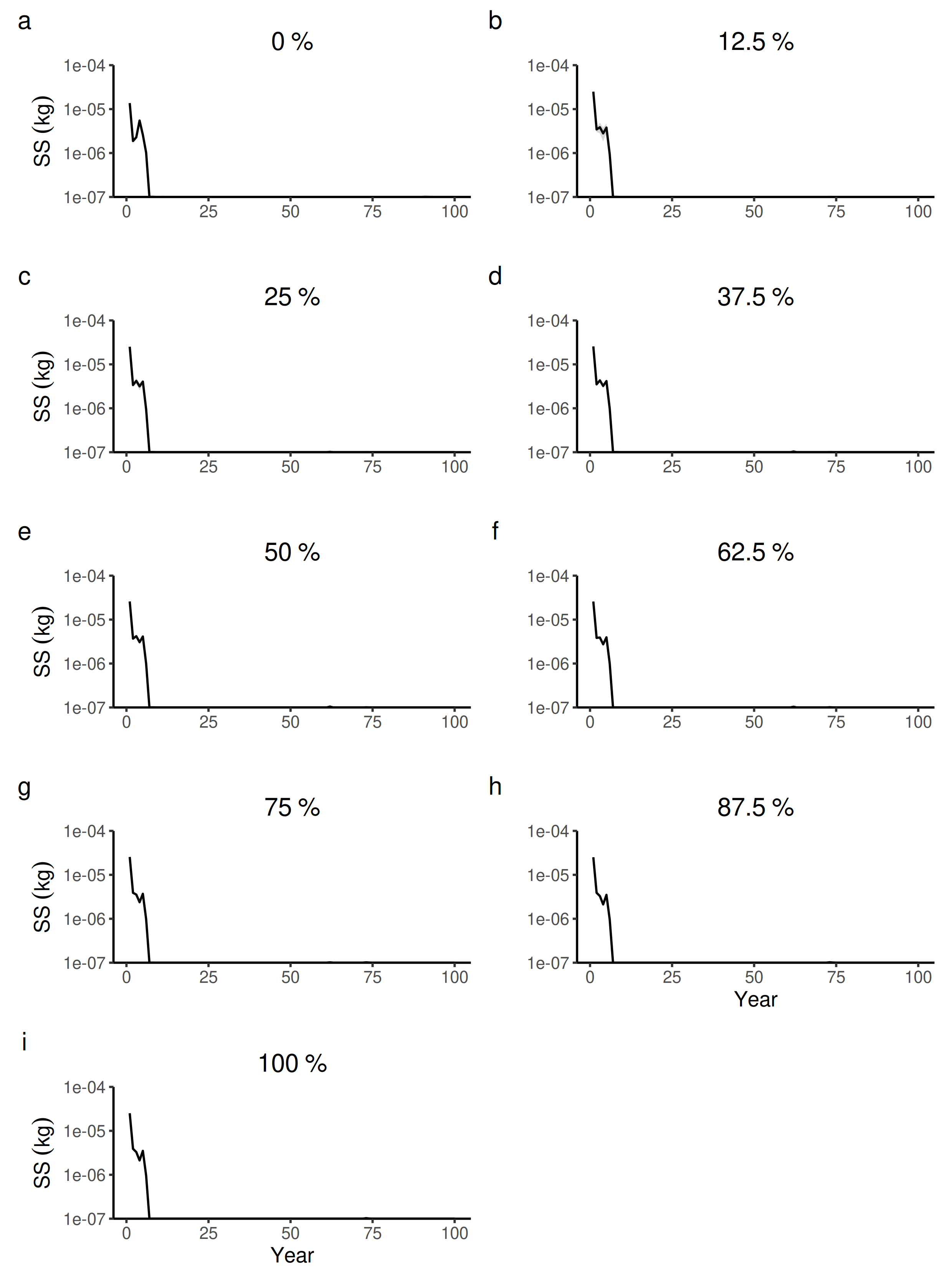

Supplement: Supplemental Information 16 [file peerj-11-14731-s016.png]

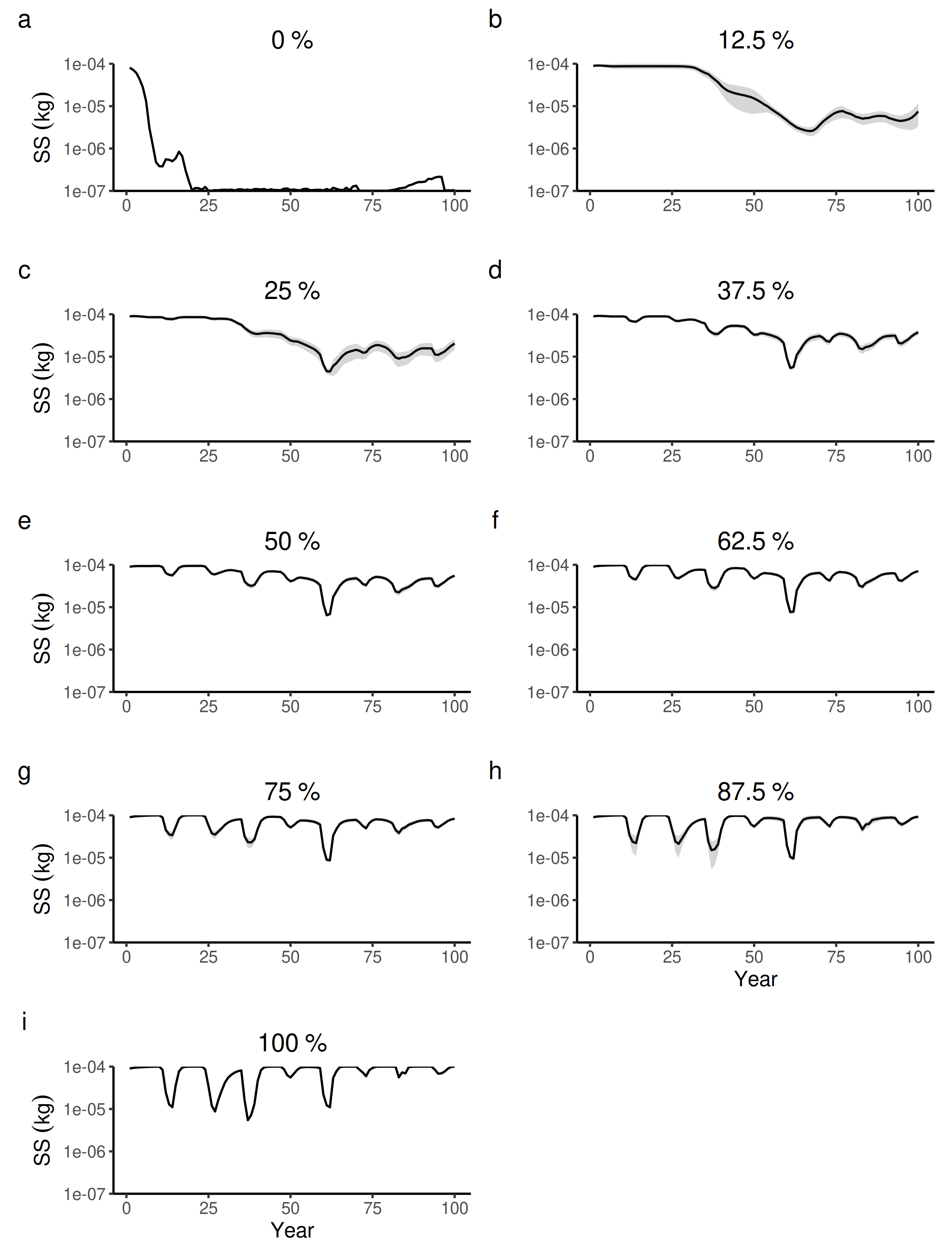

Supplement: Supplemental Information 17 [file peerj-11-14731-s017.png]

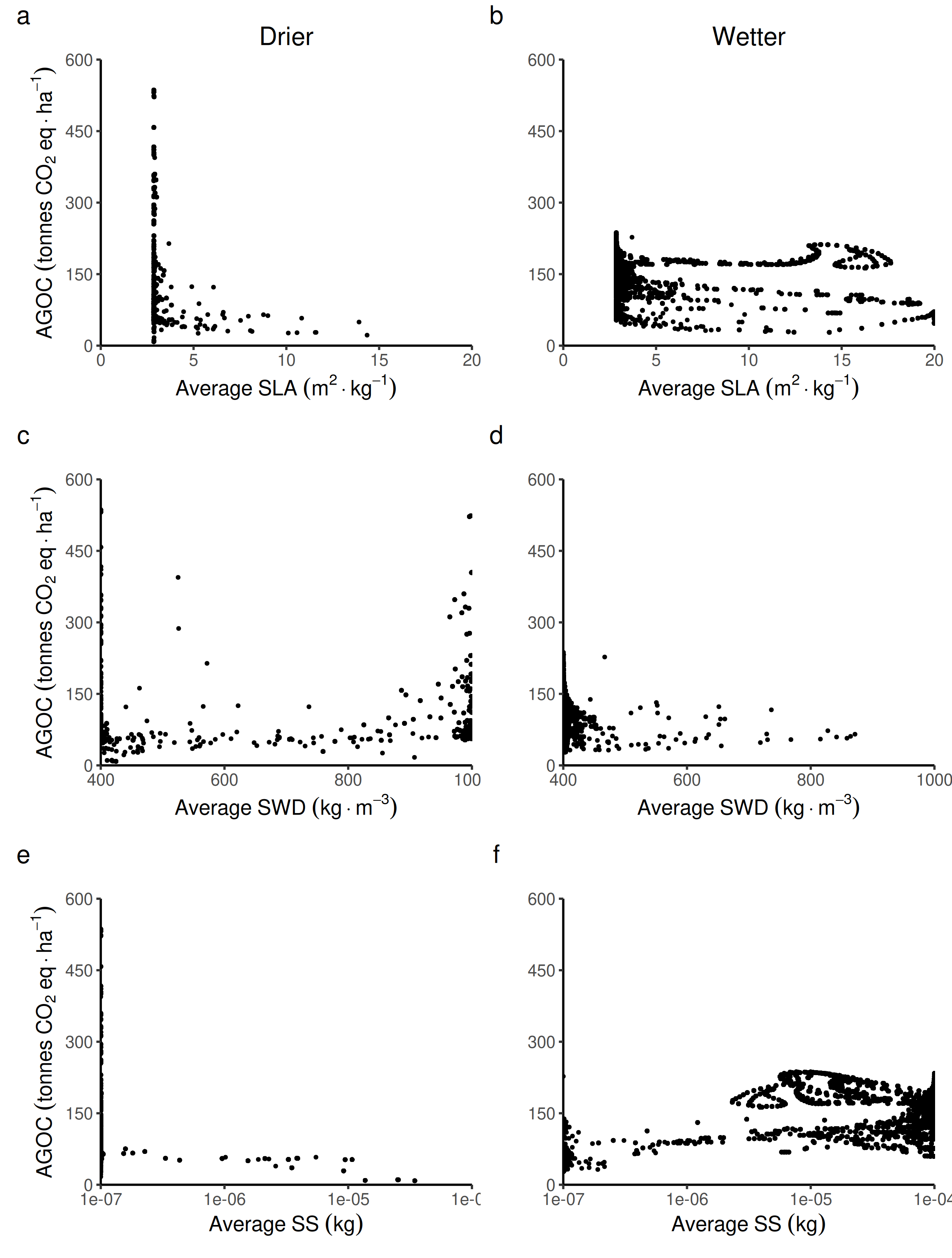

Supplement: Supplemental Information 18 — Specific leaf area: SLA, specific wood density: SWD, seed size: SS in both wetter and drier forest commons containing eight species. [file peerj-11-14731-s018.png]

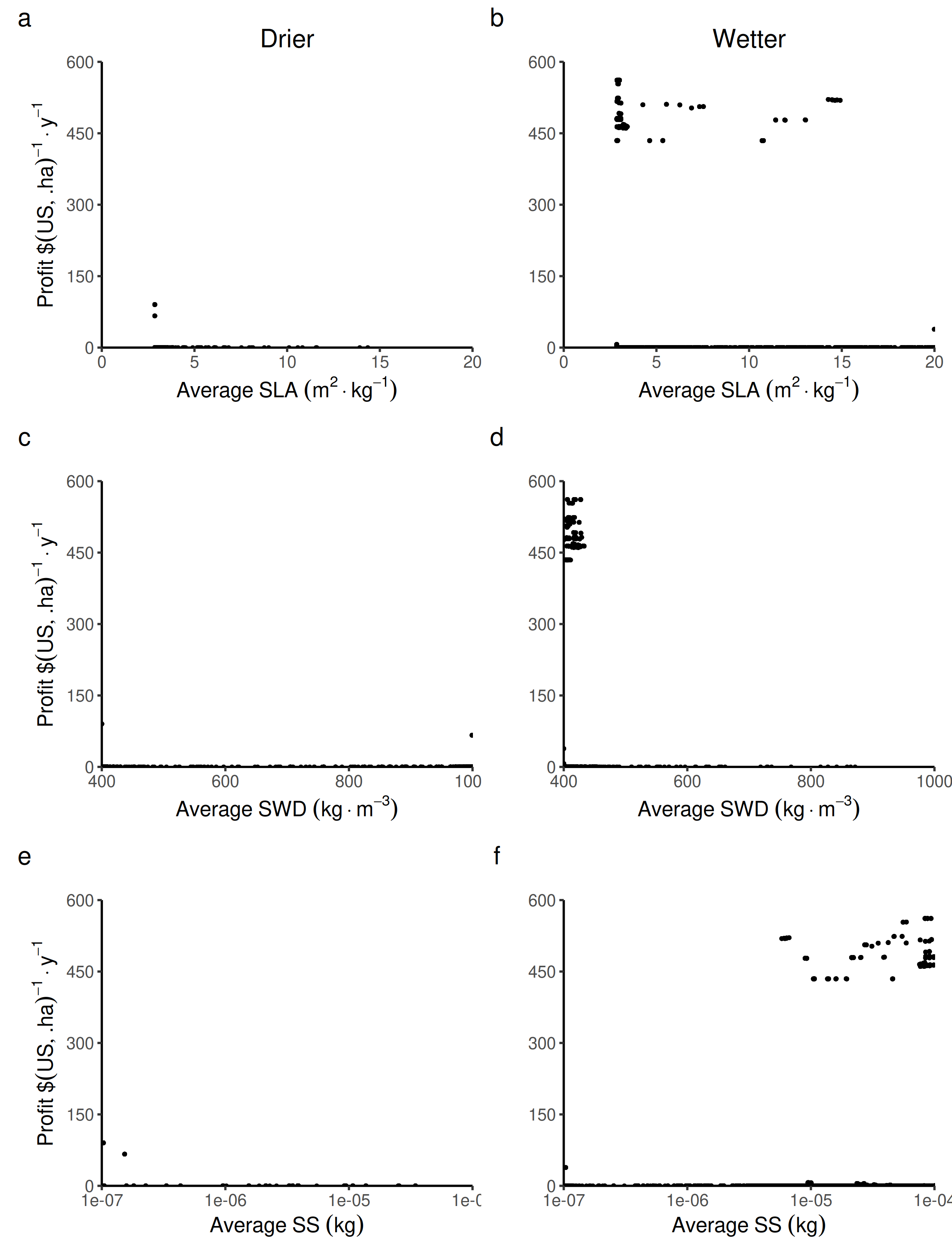

Supplement: Supplemental Information 19 — Specific leaf area: SLA, specific wood density: SWD, seed size: SS in both wetter and drier forest commons containing eight species. [file peerj-11-14731-s019.png]
